# Utilizing enantiomerically pure organic spacers for anion-centred clusters in a hybrid inorganic–organic lead-halide crystal†

**DOI:** 10.1039/d5cc01101a

**Published:** 2025-06-19

**Authors:** Markus W. Heindl, Joachim Ballmann, Felix Deschler

**Affiliations:** a Physikalisch-Chemisches Institut, Universität Heidelberg, Im Neuenheimer Feld 229 D-69120 Heidelberg Germany deschler@uni-heidelberg.de; b Anorganisch-Chemisches Institut, Universität Heidelberg, Im Neuenheimer Feld 276 D-69120 Heidelberg Germany na150@uni-heidelberg.de

## Abstract

In this work, we present structural data of the novel, chiral material (*S*-2AH)_4_[Pb_2_Br_7_]Br obtained from single crystal X-ray diffraction (SCXRD). This quasi-2D material displays anion-centred clusters within its organic layer, without the need for bi-functional spacers. Instead, the structure is based on the steric properties of the used enantiomerically pure precursors.

## Abstract 2

We report the synthesis and structure of the novel, chiral material (*S*-2AH)_4_[Pb_2_Br_7_]Br with a layered structure. Through the use of enantiomerically pure precursors, bromide-centered clusters within the non-polar, organic spacer layer are formed.

## Graphical abstract

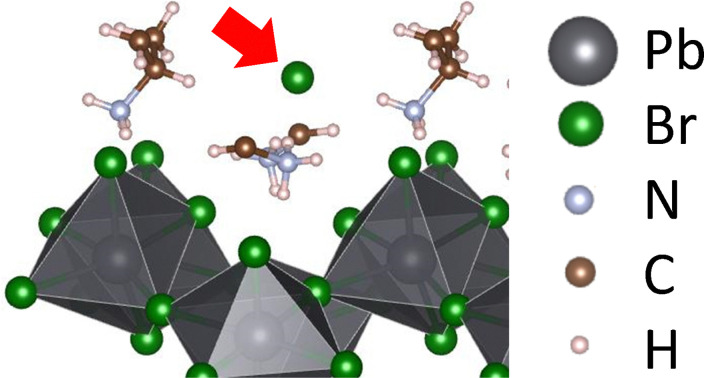

Over recent years chiral lead-halide perovskites have evolved from a structural curiosity into a key point of interest for material science.^1^ They can be fabricated through relatively simple, solution-based strategies while also displaying sought-after optoelectronic properties. These include the preferred absorption and emission of one form of circularly polarized light as well as creating spin-polarized electrical currents *via* the chirality-induced spin selectivity (CISS) effect.^1–6^ Particularly their use for the transfer of spin-polarized charge carriers has become a focus point of research over the past few years.^7–9^ Recent studies have suggested that these phenomena originate from slight structural distortions that occur when enantiomerically pure organic spacers are used instead of a racemic mixture.^10–12^ However, as a less discussed aspect, these alterations can also be much more pronounced.^13^ This opens an opportunity for the design of novel materials by utilizing the specific steric properties of pure enantiomers compared to their racemic mixtures. In this work, we will demonstrate the use of (*S*)-2-aminoheptane (*S*-2AH) to form a novel 2D-structure that allows for the incorporation of anion-centred clusters within the organic layer.

For this we add 0.18 g of PbBr_2_ (0.49 mmol, Sigma-Aldrich, ≥98%) into 2 mL of concentrated hydrobromic acid (Sigma-Aldrich, 48%) and cool the mixture in an ice-bath before adding dropwise 0.1466 mL of (*S*)-2-aminoheptane (0.97 mmol, Sigma-Aldrich, 99%). The resulting mixture is then placed in an autoclave and heated to 90 °C. After 12 h it is then cooled back to room temperature at a rate of −1 °C h^−1^. The result are clear, needle-shaped crystals at a yield of 14%.

Analysis with SCXRD reveals a layered structure composed of inorganic lead-bromide sheets separated by organic spacers (see Fig. 1). This material's unit cell has the elemental composition C_112_H_288_Br_32_N_16_Pb_8_ but is perhaps better described as (*S*-2AH)_4_[Pb_2_Br_7_]Br due to its composition out of units of two face-sharing octahedra and the isolated bromide anion within the organic layer. Further, due to the low-density packing of the spacer molecules, some carbon atoms of the heptyl chain were found to be disordered without affecting the stereogenic centre at the 2-position (see Fig. S1, ESI†). The material crystallizes in the polar non-centrosymmetric Sohncke space group *P*2_1_2_1_2_1_ and is therefore chiral (Flack *x*: −0.048(16), Hooft *y*: −0.034(4), Parson's *q*: −0.056(5), Bijvoet pairs coverage: 0.98 with P2(true) = 1.000, see ESI† for details).^1^ Purity is confirmed by combustion analysis (see Table S2, ESI†).

**Fig. 1:**
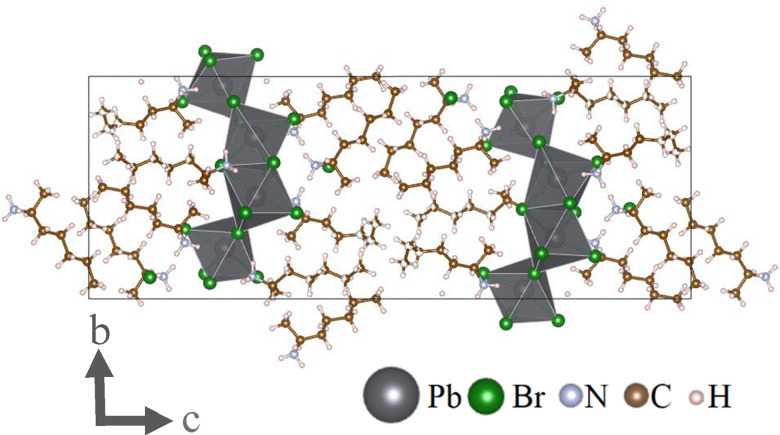
The unit cell of (*S*-2AH)_4_[Pb_2_Br_7_]Br consist of two inorganic layers made up of lead-bromide octahedra. These layers are separated by organic spacers in which a bromide anion is located. Disordered atoms omitted for clarity. This graphic was created using the VESTA software package.^14^

While the crystal's inorganic layers consist of lead-bromide octahedra, these are not arranged in one of the more established patterns, two of the best known of which perhaps are 2D networks of corner-linked octahedra (commonly known as 2D perovskites) and 1D chains of face-linked octahedra.^13,15^ Instead, the inorganic layers alternate between these two structural motives. The result are units of two face-linked octahedra that are each bound to four similar pairs to form a net-like structure (see Fig. 2a). This system is stabilized by the cationic −NH_3_^+^ moiety of one organic spacer that is located in the centre of each hole. It is also worth mentioning that – unlike a classic quasi-2D perovskite – the inorganic layers are not planar. Instead, each face-sharing octahedra pair is alternatingly sticking out above or below a virtual plane as seen in Fig. 2b. We have hence chosen to refer to this two-dimensional net-like structure as “quasi-2D”, to account for this apparent roughness. From this representation it is also apparent that there is another bromide ion that is not part of any octahedron, nor directly linked to the inorganic layer. Instead, this anion is located inside the nonpolar organic spacer region.

**Fig. 2:**
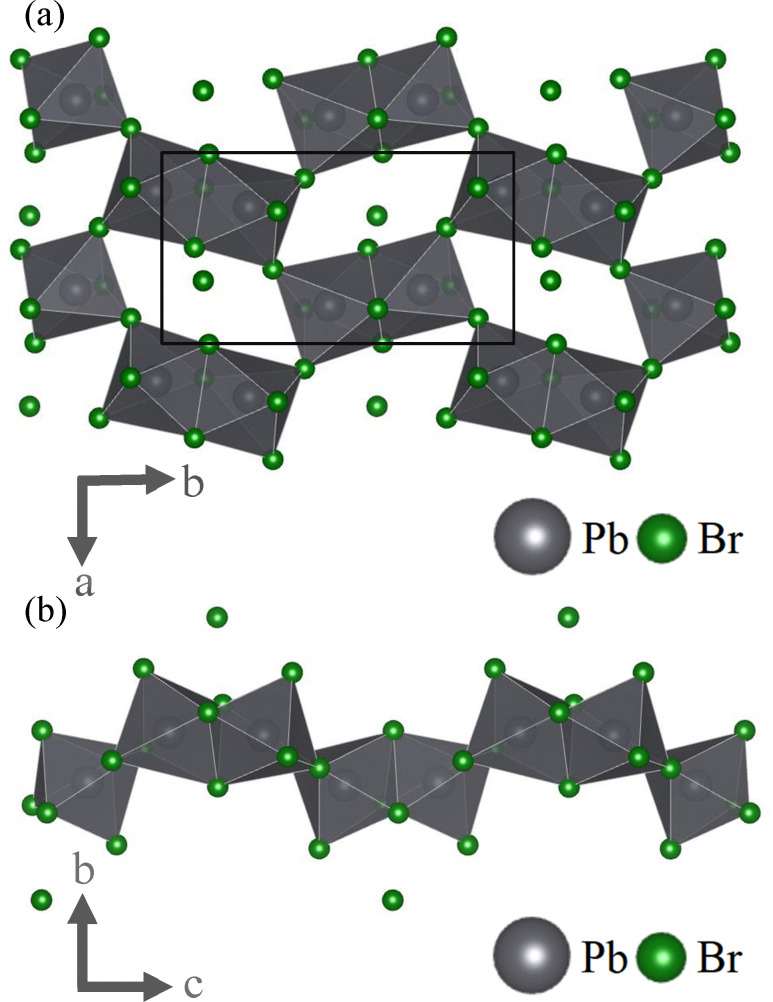
Inorganic components of (*S*-2AH)_4_[Pb_2_Br_7_]Br displayed without the organic spacers, as viewed along the (a) *c*- and the (b) *a*-axis. The inorganic layer is composed out of units of two face-sharing octahedra which in turn are connected through shared corners, forming a net-like structure. Additional bromide-ions are located above or below the resulting holes of the inorganic network. Black lines in figure (a) indicate an exemplary unit cell. This graphic was created using the VESTA software package.^14^

Looking at this odd bromide ion, we find that it is surrounded by four positive charges in the form of protonated amino groups −NH_3_^+^ of the organic spacer cations located between this anion and the inorganic layer. However, this bromide can only form hydrogen bonds with three of these protonated amino groups at the respective distances of 2.39653(4) Å, 2.45616(7) Å and 2.46708(4) Å, resulting in a distorted trigonal pyramid. The fourth protonated amino group is facing in the opposite direction, while the respective molecule's nonpolar tail is placed between the two, resulting in a much greater distance of over 5 Å, too large to be considered a potential hydrogen-bond. This protonated amino group hence binds to the neighbouring interlayer bromide ion. This particular structure can only form since all relevant protonated amino groups are facing towards the same side of their respective alkyl groups (see Fig. 3). This is a strong indication that this is the direct result of the use of enantiomerically pure organic precursors, since otherwise the cationic group would be oriented randomly towards either side of the carbon chain, or the alkyl-group would be forced to take a position that is sterically more demanding. Either option is likely not energetically favourable. Indeed, if the synthesis is repeated with a racemic mixture of 2-Aminoheptane, no single crystals are formed. There are however literature reports that suggest the existence of a classic 2D lead-bromide perovskite utilizing (*rac*)-2-Aminohaptane as spacer, indicating that the structure presented here is likely not formed under the tested fabrication conditions when using racemic precursors.^16^ It is further worth noting that – as seen in Fig. 3 – all protonated amino groups binding to the bromide ion are located between the inorganic layer and said ion. This means that there is no transition mediated by covalently bound polarized groups between this ion and the nonpolar layer as usually is the case for low-dimensional hybrid materials. Instead, we observe a more direct interface between polar and nonpolar components.

**Fig. 3:**
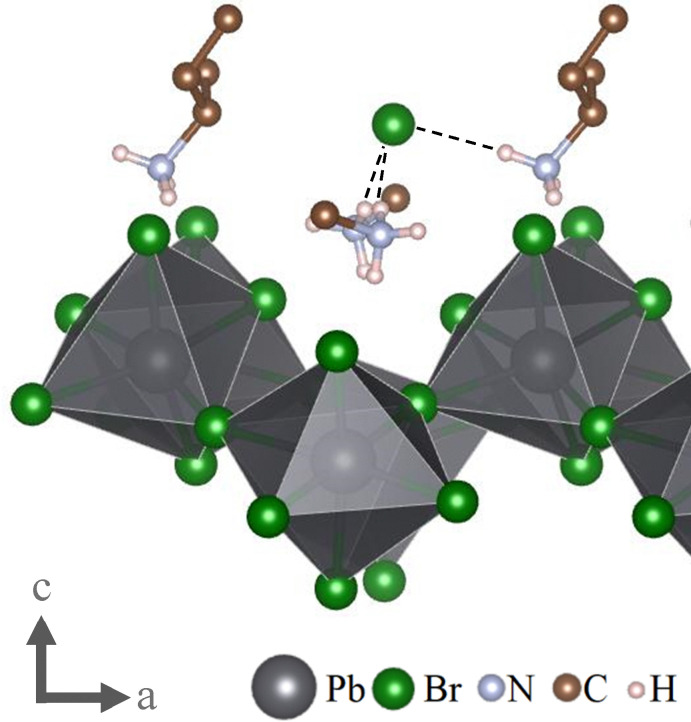
The interlayer bromide ion with the four surrounding –NH_3_^+^-moieties. While three of these protonated amino groups form hydrogen bonds with the central anion, the fourth (left) faces away relative to its connected alkyl-group. Further, all protonated amino groups are located between the inorganic layer and the central bromide anion, creating a direct interface between the bromide anion and the organic layer. Dashed lines indicate hydrogen bonds. Parts of the organic spacers were omitted for clarity. This graphic was created using the VESTA software package.^14^

The observation of this bromide ion within the organic layer reminds of recent reports by Aubrey *et al.* and a previous study by Mercier and Riou who each reported the synthesis of single crystals made up of alternating layers of perovskite sheets and other inorganic layers or clusters separated through bi-functional organic spacers, thereby potentially opening up a new strategy for the design of functional materials.^17,18^ This approach has already been successfully demonstrated to add antiferromagnetic coupling effects to an otherwise diamagnetic perovskite-like lead-halide material by incorporating transition-metal clusters into the crystal structure.^19^ Others have found that this material class can display increased resistance towards air and moisture and have pointed out its anisotropic charge transport properties.^20^ However, our material differs in a few key points. First, the structure presented here still possesses a van der Waals gap since the individual layers are not linked through covalent bonds. This may enable quantum confinement effects.^21^ Second, while in other structures the non-perovskite layer or cluster usually forms though the direct interaction of the organic spacers or is centred around one or multiple (metal-) cations, here the inorganic heterostructure is centred around a bromide anion, making this – to the best of our knowledge – the first reported anion-centred interlayer cluster. Further, unlike previously reported materials displaying interlayer clusters, (*S*-2AH)_4_[Pb_2_Br_7_]Br is chiral and could hence be utilized for chiroptic and spintronic applications.^1^ Finally, while previous works have relied on bi-functional organic spacers to create these kinds of heterostructures, the material presented here merely relies on the steric properties of mono-functional (*S*)-2-aminoheptane. We therefore believe that this crystal structure represents an interesting addition to the pool of materials with interlayer clusters.

To summarize, we report the novel, chiral material (*S*-2AH)_4_[Pb_2_Br_7_]Br that combines structural motives of well-established 1D and 2D organic–inorganic hybrids often studied in regards to their optoelectronic properties. This material displays unusual anion-centred clusters within its organic layers which are not a result of bi-functional spacers but rather originate from the specific steric properties of the enantiomerically pure precursors used. We believe that this material is a promising candidate for an in-depth investigation of its polar-nonpolar interfaces and the resulting structural effects and properties as for instance piezoelectricity. Further, we hope that this report will help to promote the research on the use of enantiomerically pure precursors in organic–inorganic hybrid materials beyond chiroptic properties and give fresh impulses for the investigation of interlayer clusters.

M. W. H. synthesized the material, evaluated and visualized results and drafted the manuscript. J. B. performed and analysed SCXRD measurements. F. D. supervised the project and guided its direction. All authors reviewed the manuscript and contributed their ideas to its final form.

This project has received funding from the European Research Council (ERC Starting Grant agreement no. 852084-TWIST).

## Conflicts of interest

There are no conflicts to declare.

## Supplementary Material

CC-061-D5CC01101A-s001

## Data Availability

All experimental data, and detailed experimental procedures are available in the ESI.† SCXRD data is available under CCDC 2425604.
