# Supplementary material for: Utilizing enantiomerically pure organic spacers for anion-centred clusters in a hybrid inorganic–organic lead-halide crystal
Source: Chem Commun (Camb). 2025 Jun 19;61(58):10855–8. doi: 10.1039/d5cc01101a (PMC12178114; doi:10.1039/d5cc01101a)
Supplement: CC-061-D5CC01101A-s001 [file CC-061-D5CC01101A-s001.pdf]

Supplementary Information (SI) for ChemComm.
This journal is © The Royal Society of Chemistry 2025

Supporting Information - Utilizing enantiomerically pure organic spacers for anion-centred clusters in a hybrid inorganic-organic lead-halide crystal

Markus W. Heindl, Joachim Ballmann, Felix Deschler

X-Ray Crystal Structure Determinations

Crystal data and details of the structure determination are compiled in Table S1. Full shells of intensity data were collected at 120(1) K with an Agilent Technologies Supernova-E CCD diffractometer (Cu-K_α radiation, microfocus X-ray tube, multilayer mirror optics). Detector frames (typically ω-, occasionally φ-scans, scan width 1.0°) were integrated by profile fitting.[1] Data were corrected for air and detector absorption, Lorentz and polarization effects[2,3] and scaled essentially by application of appropriate spherical harmonic functions.[4,5,6] Absorption by the crystal was treated numerically (Gaussian grid).[6,7] An illumination correction was performed as part of the numerical absorption correction.[6]

Using OLEX2,[8] the structure was solved with SHELXT[9] (intrinsic phasing) and refined with SHELXL[10] by full-matrix least squares methods based on F^2 against all unique reflections. All non-hydrogen atoms were given anisotropic displacement parameters. Hydrogen atoms were generally input at calculated positions and refined with a riding model.[11] Split atom models were used to refine disordered groups. When found necessary, suitable geometry and adp restraints were applied.[12]

CCDC 2425604 contains the supplementary crystallographic data for this paper. These data can be obtained free of charge from the Cambridge Crystallographic Data Centre's and FIZ Karlsruhe’s joint Access Service via https://www.ccdc.cam.ac.uk.

Table S1: Crystallographic information for $(S\text{-}2AH)_4[Pb_2Br_7]Br$.

Empirical formula	$C_{28}H_{72}Br_8N_4Pb_2$
Formula weight	1518.55
Temperature [K]	120(1)
Crystal system	*orthorhombic*
Space group (number)	$P2_12_12_1$ (19)
a [Å]	8.1442(2)
b [Å]	14.8830(4)
c [Å]	40.3060(8)
α [°]	90
β [°]	90
γ [°]	90
Volume [Å^3]	4885.5(2)
Z	4
ρ_{calc} [gcm^{-3}]	2.065
μ [mm^{-1}]	21.063
F(000)	2848
Crystal size [mm^3]	0.05×0.11×0.17
Radiation	Cu K_α (λ=1.54184 Å)
2θ range [°]	6.33 to 143.84 (0.81 Å)
Index ranges	$-9 \le h \le 9$<br>$-18 \le k \le 18$<br>$-49 \le l \le 49$
Reflections collected	102412
Independent reflections	9367 (R_{int} = 0.1104)
Completeness to θ = 67.684°	100.0 %
Data / Restraints / Parameters	9367 / 244 / 466
Absorption correction T_{min}/T_{max} (Method)	0.1090 / 0.5420 (Gaussian grid)
Goodness-of-fit on F^2	1.073
Final R indexes [$I \ge 2\sigma(I)$]	R_1 = 0.0658<br>wR_2 = 0.1752
Final R indexes [all data]	R_1 = 0.0694<br>wR_2 = 0.1800
Largest peak/hole [eÅ^{-3}]	1.82/−1.66
Hooft / Parson's /Flack parameter	-0.034(4) / -0.056(5) / -0.048(16)
Extinction coefficient	0.00031(3)
CCDC number	2425604

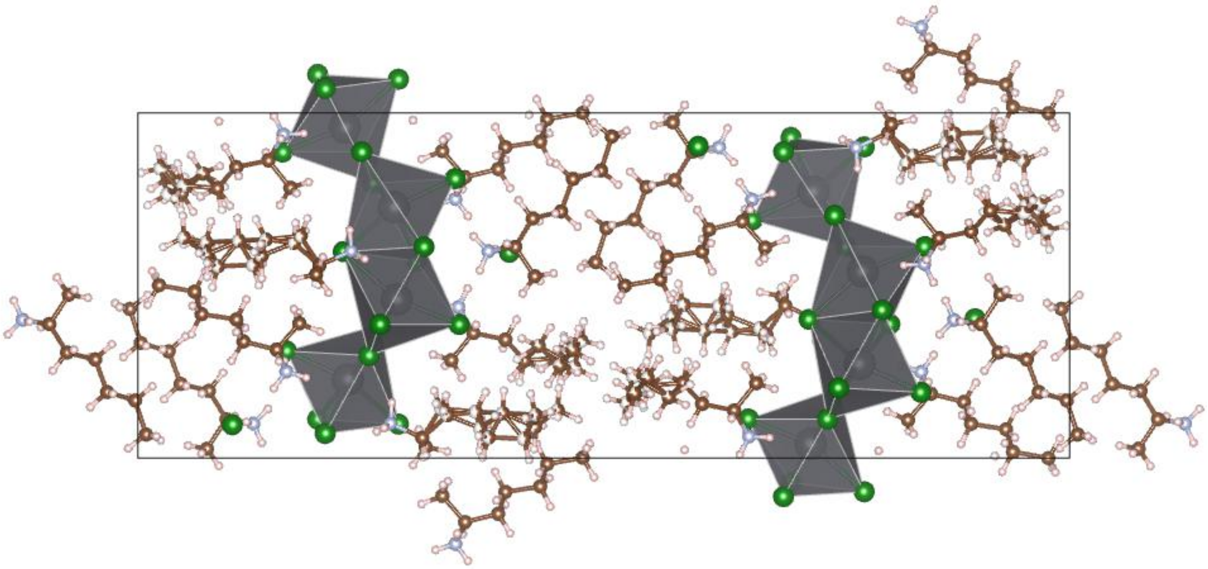

Figure S1: Complete crystal structure of (S-2AH)$_4$[Pb$_2$Br$_7$]Br, including disordered atoms. Disorder is limited to the organic layer. Two out of four organic spacers (per formula unit) were disordered over two positions each. Free variables that add up to an occupancy of 1.0 for each disordered atom were used to modelling the structure.

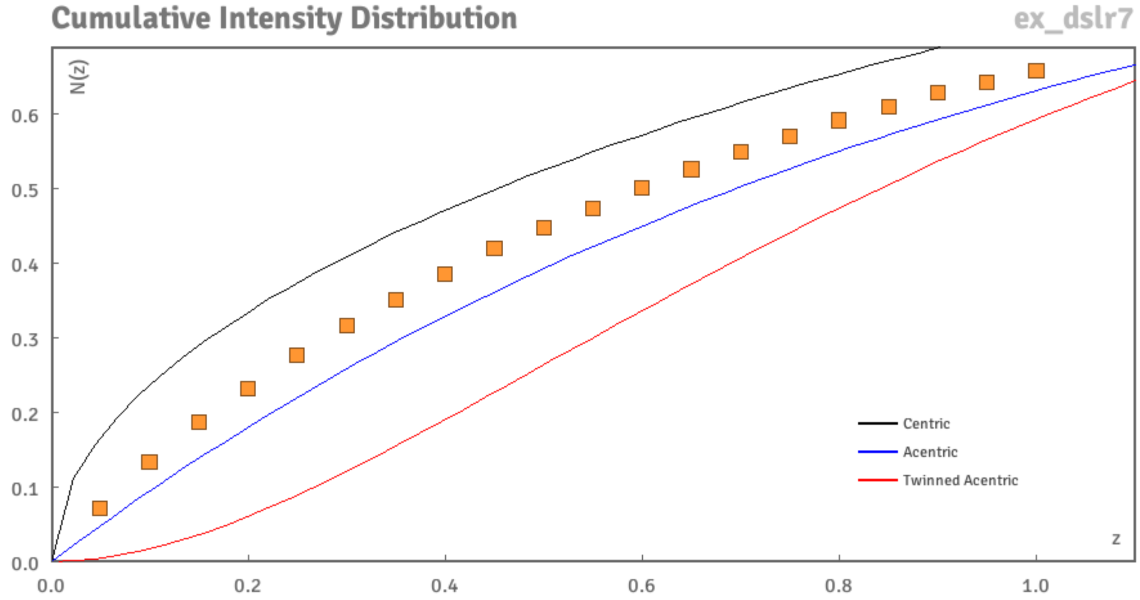

Figure S2: Cumulative intensity distribution for crystals of (S-2AH)$_4$[Pb$_2$Br$_7$]Br, indicative of a non-centro-symmetric space groups (orthorhombic $P2_12_12_1$).

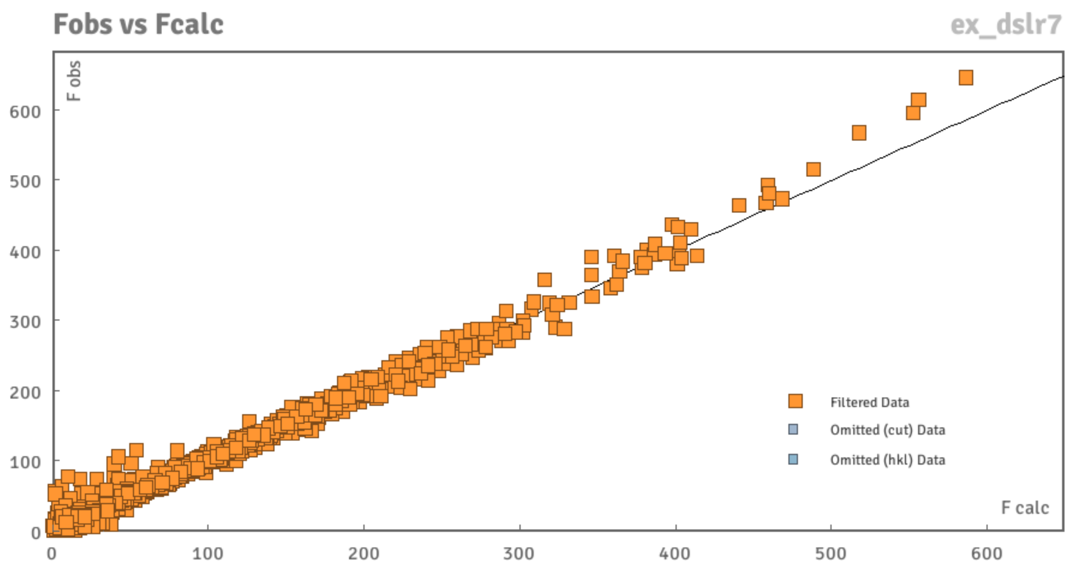

Figure S3: F_{obs} vs. F_{calc} plot for the analysed SCXRD data. The Goodness-of-fit on F^2 is determined as 1.073, the final R indexes [I>=2σ (I)] as R1 = 0.0658.

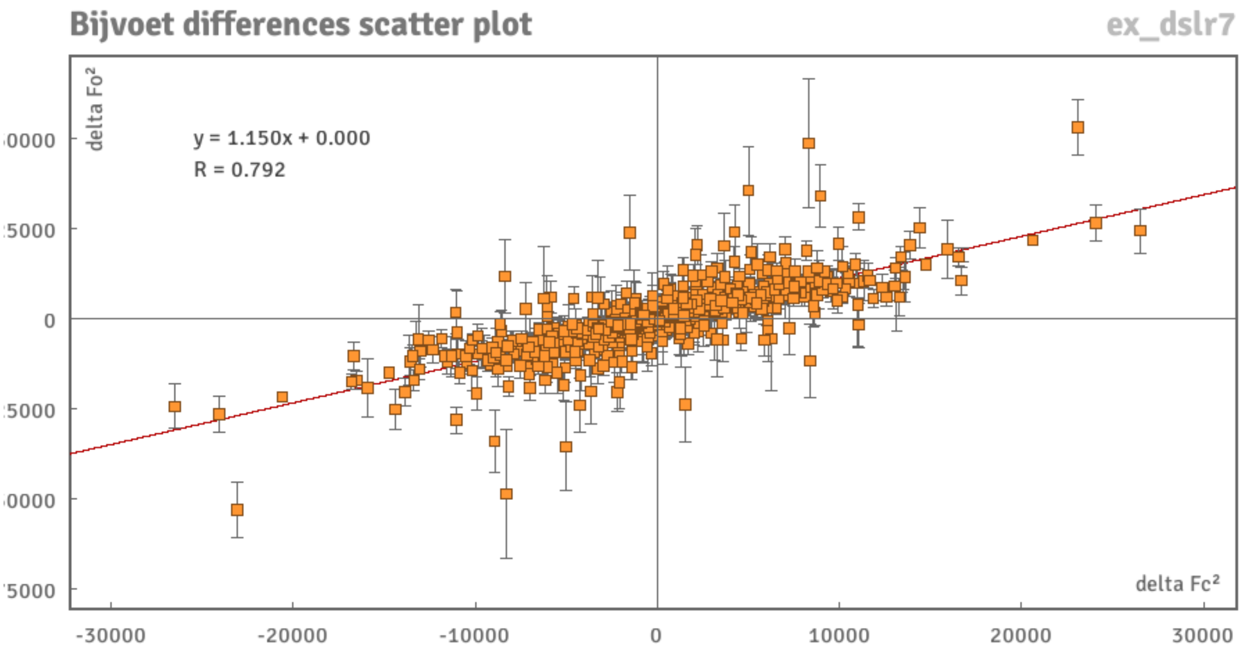

Figure S4: Bijvoet differences scatter plot (Bijvoet pair analysis using Gaussian distribution: Bijvoet pairs (all): 4069, Bijvoet pairs (used): 4069, Bijvoet pairs coverage: 0.98, G: 1.069(9, P2(true): 1.000, P2(false): 0.000e+00, P3(true): 1.000, P3(false): 0.000e+00, P3(racemic twin): 0.000e+00, correlation coefficient: 0.9978).

Table S2: Combustion analysis of as-synthesized $(S\text{-}2AH)_4[Pb_2Br_7]Br$ yields the following results

Element	Calculated (%)	Measured (%)
H	4,78	4,97
C	22,15	22,39
N	3,69	3,62

References

(1) Kabsch, K., in *International Tables for Crystallography*, Eds. Rossmann, M. G.; Arnold, E., *Vol. F*, Ch. 11.3, Kluwer Academic Publishers, Dordrecht, The Netherlands, **2001**

(2) SAINT, Bruker AXS GmbH, Karlsruhe, Germany **1997**-**2013** and SAINT V8.40A, Bruker AXS Inc., Madison, Wisconsin, USA, **2018**.

(3) *CrysAlisPro*, Rigaku Oxford Diffraction, Rigaku Polska Sp.z o.o., Wrocław, Poland **2015**-**2024**.

(4) Blessing, R. H., *Acta Cryst.* **1995**, *A51*, 33-38.

(5) Sheldrick, G. M., *SADABS*, Bruker AXS GmbH, Karlsruhe, Germany **2004-2014**.

(6) *SCALE3 ABSPACK, CrysAlisPro*, Rigaku Oxford Diffraction, Rigaku Polska Sp.z o.o., Wrocław, Poland **2015**-**2024**.

(7) Busing, W. R.; Levy, H. A., *Acta Cryst.* **1957**, *10*, 180-182.

(8) Dolomanov, O. V.; Bourhis, L. J.; Gildea, R. J.; Howard, J. A. K.; Puschmann, H., *J. Appl. Cryst.* **2009**,*42*, 339-341.

(9) (a) Sheldrick, G. M., SHELXT, University of Göttingen and Bruker AXS GmbH, Karlsruhe, Germany, **2012**-**2018**; (b) Sheldrick, G. M., *Acta Cryst.* **2015**, *A71*, 3-8.

(10) (a) Sheldrick, G. M., *SHELXL-20xx*, University of Göttingen and Bruker AXS GmbH, Karlsruhe, Germany **2012**-**2018**; (b) Sheldrick, G. M., *Acta Cryst.* **2008**, *A64*, 112-122; (c) Sheldrick, G. M., *Acta Cryst.* **2015**, *C71*, 3-8.

(11) (a) Rollett, J. S. in *Crystallographic Computing*, Eds. Ahmed, F. R.; Hall, S. R.; Huber C. P., Munksgaard, Copenhagen, Denmark, p. 167, **1970**; (b) Watkin, D. in *Crystallographic Computing 4*, Eds. Isaaks, N. W.; Taylor, M. R., Ch. 8, IUCr and Oxford University Press, Oxford, UK, **1988**; (c) Müller, P.; Herbst-Irmer, R.; Spek, A. L.; Schneider, T. R.; Sawaya, M. R., in *Crystal Structure Refinement*, Ed. Müller, P., Ch. 5, Oxford University Press, Oxford, UK, **2006**; (d) Watkin, D., *J. Appl. Cryst.* **2008**, *41*, 491-522.

(12) Thorn, A.; Dittrich, B.; Sheldrick, G. M., *Acta Cryst.* **2012**, *A68*, 448-451.
